# Developing a National Minimum Data Set for Kawasaki Disease Registry in Iran

**DOI:** 10.3389/fped.2022.834306

**Published:** 2022-02-28

**Authors:** Zainab Qazizadeh, Leila Shahbaznejad, Mohammad Reza Navaeifar, Mohammad Sadegh Rezai

**Affiliations:** ^1^Mazandaran Population-Based Cancer Registry, Mazandaran University of Medical Sciences, Sari, Iran; ^2^Pediatric Infectious Diseases Research Center, Communicable Diseases Institute, Mazandaran University of Medical Sciences, Sari, Iran

**Keywords:** minimum data set (MDS), registry, mucocutaneous lymph node syndrome (Kawasaki disease), Iran, Kawasaki

## Abstract

**Background:**

Kawasaki Disease is an acute and self-limited systemic inflammatory and febrile illness, which is the most common cause of acquired heart disease in children in developed countries. The incidence of KD in Asian countries is high. But, data is not available from the Middle East. So, the aim of this study was to develop an MDS to set up a national registry for KD to estimate the burden of disease in Iran.

**Materials and Methods:**

This cross-sectional and descriptive study was conducted in 2020. Literature review, data collection from patients medical records, and expert panel approach were used to design this MDS. Data elements with a Content Validity Ratio (CVR) of more than 0.56 were selected as the MDS of the registry.

**Results:**

Overall, 99 data elements were recognized. Of which, 51 and 48 data elements were verified and rejected, respectively. Moreover, 17 data elements were added as required by experts. Eventually, 68 data elements were chosen as the MDS of the national KD registry of IRAN; of which, 17 and 51 data elements were classified as administrative and clinical data, respectively.

**Conclusions:**

These precise, integrated, and comprehensive developed data elements and the national KD registry will lead to effective disease management and thus, improve the quality of care and, consequently, improve public health.

## Introduction

Kawasaki disease (KD), an acute and self-limited systemic inflammatory and febrile illness occurs generally in children under 5 years old (1, 2). Almost 85% of patients are <5 years of age with an average of ~2 years. However, some cases have been reported in young and old people (3). Fever, rash, cervical lymphadenopathy, conjunctivitis, oral changes, extremity changes, skin and mucous membrane infection are its major symptoms (2, 4). It can seriously affect the heart and its coronary arteries (5, 6). Almost all deaths in patients with KD are due to secondary heart disease or its consequences such as myocardial infarction (MI), chest pain, arrhythmia, and sudden death (5).

KD is the most common cause of acquired heart disease in children in developed countries (2). The pathogenesis and etiology of Kawasaki are still uncertain (7). Based on the presence of familial clusters and the increased incidence (near 10 times) in the Asian population, a strong genetic origin has been proposed (4, 7). Diverse epidemiological reports indicate that the incidence of KD is increasing significantly in developing and industrializing countries like India, China, and Latin America (6). The incidences of KD in Asian countries, particularly in Northeast Asia, are meaningfully higher than those in the United States and Europe (8). Japan, South Korea, and Taiwan are the three Asian countries that have reported an ongoing increase in KD incidence (9–11). KD is most common in Japan. The incidence in Japan is ~240/100 000 children under 4 years old (12). By 2014, ~300 000 KD patients have been registered in this country (9). The incidence in Korea, the second-highest worldwide, is 134.4 cases per 100 000 for children <5 years old (10). The incidence rate of KD has gradually increased in most other Asian countries (13). Unfortunately, incidence data is not available from the Middle East. A limited number of patients have been reported from this region's countries, most of them coming from Turkey and Iran (13–16).

Since KD is a major cause of acquired heart disease in children, and due to the sudden death because of coronary aneurysms and thrombosis, effective management of KD is significantly beneficial for public health. Active supervision and management could potentially lead to long-term benefits for patients, practitioners, and society by facilitating the recognition, prevention, and treatment of KD cases. It also yields cost reduction at the national level (5). It has proved that setup a disease registry is effective in reducing morbidity and mortality in the population (17).

The registry is a database that uses minimum data set (MDS) to accurately analyze the burden of disease (17, 18). Identifying and designing MDS is a preliminary and important step to achieve the goals of the registration program, which leads to the improved disease control program (19).

The aim of the MDS is to determine the data elements that need to be gathered and to develop a database as a comprehensive source of precise information for decision-makers and policymakers (20). Moreover, MDS enables measuring the result of secondary prevention programs (21).

There are some issues in Iran related to the implementation of diseases include data elements duplication in healthcare forms, lack of unified health information systems, and eventually, lack of national standard minimum data sets (22). Given that effective management of KD depends on the existence of registration programs in order to improve diseases control programs; nevertheless, a uniform standard tool for collecting KD data has not yet been developed in Iran. The purpose of this study was to develop a national MDS for creating a KD national registry in Iran.

## Materials and Methods

**Figure 1:**
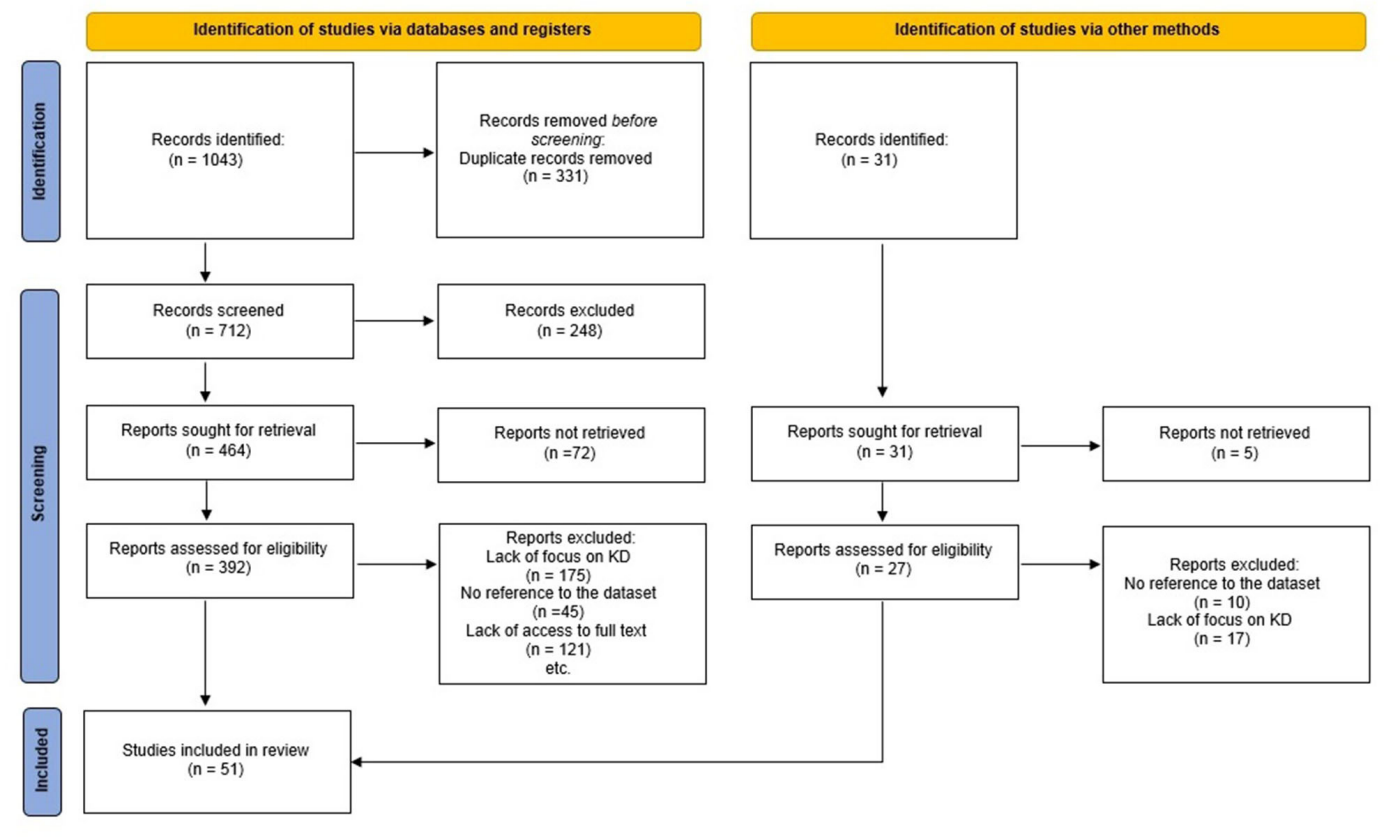
PRISMA flow diagram for the systematic reviews.

This cross-sectional and descriptive study was conducted in 2020. Literature review, data collection from patients medical records, and expert panel approach were used to design this MDS. In the first phase, a literature review was carried out to specify all possible data elements to be included in the final minimum data set. Articles, forms, guidelines, and reports on the internet were reviewed. We carried out a systematic review of ISI Web of Science, PubMed, Scopus, Google Scholar, and Persian databases to recognize all References related to the MDS of KD which were published until February 2020 (Figure 1). We used a search strategy using Medical Subject Headings (MeSH) and free terms allied to mucocutaneous lymph node syndrome, Kawasaki disease, minimum data set, datasets, common data elements, database, and registries. We scanned all references of retrieved articles, as complementary searches, to extract other probable data sources. All other related studies were retrieved.

The search strategy was limited to English and Persian languages. The included criteria were full-text articles, guidelines, forms, and reports from reliable sources published until February 2020. Other types of resources including abstracts without full-text, non-peer-reviewed articles, letters to editors, short communication, even forms and reports from weblogs were excluded. Then, data elements were evoked and recorded into a checklist. In addition, data was collected from patients medical records based on the International Classification of Diseases-10th Revision (ICD-10) related to KD. The final checklist included data elements got from the literature review and patients' medical records. Data elements were split into two categories: administrative and clinical.

In the next phase, an expert panel was held to appraise and specify the MDS of KD. Ten pediatric specialists with more than 5 years of experience in KD treatment from different universities of medical sciences participated in this panel. We sent the aim of the study, the process of identifying MDS, and the extracted MDS to all experts, 2 weeks before starting the panel. Experts' initial opinions on the data elements were collected and calculated using the Delphi method. Content Validity Ratio (CVR) of more than 0.62 was considered as a condition of expert consensus on the acceptance of data elements as the final MDS for the KD national registry. Participants were also asked to propose any additional comments on desired MDS such as other data elements that were necessary for the KD registry according to their opinion. After collecting all comments and calculating CVR for each element, we held a focus group and brainstorming sessions for discussing elements which either were scored <0.62 or were proposed by experts. Due to the prevalence of COVID-19 disease, sessions were conducted using Skype software. Sessions continued until panel members reached a consensus to determine the final data elements. After discussion on each identified data element, at the end of the third session, by sending the finalized data elements to each member of the panel, they were asked to announce their decisive agreement with the selected elements to the secretary of the panel in case of final confirmation.

## Results

The result of the systematic review showed despite the importance of KD, there is no specific registration program for KD in the world. All countries report their statistics based on the cross-sectional surveys, mostly annual, of hospital admission and discharge data. Only some KD case report forms were found in the United States, Korea, and Hong Kong. As a result, in order to develop the minimum data set for the national KD registry in Iran, we used these forms along with the guidelines for diagnosis, treatment, and management of KD found in different countries and the extracted data elements from medical records of patients in pediatric hospitals in Iran.

Overall, 99 data elements were recognized. Of which, 51 and 48 data elements were verified and rejected, respectively. Moreover, 17 data elements were added as required by experts. Eventually, 68 data elements were chosen as the MDS of the national KD registry of IRAN entitled (IR-KDMDS). The final minimum data set was divided into administrative and clinical categories with 17 and 51 data elements, respectively. The demographic characteristics of the experts are shown in Table 1.

**Table 1:** The demographic characteristics of the experts.

**Participants**	**Numbers**	**Gender**		**Age**	**Experience**
		**F**	**M**	**(average)**	**(average)**
Pediatric Infectious disease specialist	5	1	5	52	25
Heart specialist	3	1	2	49	17
Rheumatologist	2	2	0	45	15

The administrative data consists of demographic data such as full name, national code, sex, and age. The clinical data elements contain patient's hospitalization data, patient's history data (including the history of allergies, history of vaccination, history of infectious disease, history of other diseases, history of the post-Kawasaki disease, history of vasculitis and autoimmune disease, history of medications, history of hospitalization, history of contact with certain detergents in a recent month, family history of KD, vasculitis and autoimmune disease), major criteria of KD, patient′s body organs review, and finally, diagnostic and therapeutic procedures including laboratory tests, other paraclinical evaluations, drug, and surgical treatments. The complete elements of IR-KDMDS are presented in Table 2.

**Table 2:** Some of administrative and clinical data elements of IR-KDMDS.

**Section**	**Sub-section**	**Example of data elements**
Demographic data		Full name, national code, father's name, date of birth, gender, address, phone number, socio-economic status
Hospitalization data		Record number, admission date, discharge date, length of stay (LOS)
History data	History of allergies	Allergy type, time interval to KD, manifestations
	History of vaccination	type of vaccine, Up-to-date vaccine, induction date, time interval to KD
	History of infectious disease	Type of disease, age at diagnosis, involved organs, time interval to KD
	History of other diseases	Type of disease, age at diagnosis, time interval to KD
	History of post-Kawasaki disease	
	History of vasculitis and autoimmune disease	
	History of medications	Drug name, duration of use, Reason, time interval to KD
	History of hospitalization	Cause (type of disease), length of stay (LOS), time interval to KD
	History of contact with certain detergents in a recent month	Detergent name, manifestations, contact time, time interval to KD
	Family history of KD, vasculitis and autoimmune disease	History of KD in the family, which relative, type of disease, age
Major criteria		Fever, conjunctivitis, cervical lymphadenopathy, change in the extremities, skin rash, changes in the lips and mouth, duration
Body organs review		Cardiovascular symptoms, gastrointestinal symptoms, renal symptoms, skin symptoms, lymphadenopathy of other parts of the body, joint symptoms, central and peripheral nervous system, respiratory symptoms, concurrent infection, vision, hearing, throat, erythema and stiffness at the BCG injection site, other symptoms, duration of each symptom
Diagnostic procedures	Laboratory test	CBC, inflammatory indexes, U/A & U/C, liver function & biochemistry, lipids profile, electrolyte, CSF, complements, autoantibodies, CD Marker, IHC, antiviral antibodies, B/C, throat swab culture, B & T cell counts, ABG, serum Ig, IgM, IgG COVID-19, RT-PCR COVID-19, examination date
	Other paraclinical evaluation	Type of paraclinical service (CXR, abdominal sonography, ECG, echocardiography, spiral chest CT Scan) findings, report images
Therapeutic procedures	Drug treatment	Name of drug, dose, unit, consumption per day, start date, cease date, side effects, reason for selection
	Surgical treatment	Date of surgery, type of surgery

## Discussion

Kawasaki disease is one of the most common childhood vasculitis that can lead to serious complications, considerable morbidity, and mortality. Proper and timely treatment of disease can prevent these complications, and this indicates the importance of early and accurate diagnosis of the disease (23). Up to now, the number of KD cases has been reported in more than 60 countries worldwide, which is growing dramatically in developing countries (8, 24). Existence of accurate information about KD can be helpful for caregivers and also determine the burden of KD on treatment settings and even the prevalence of coronary heart disease in adults in the long term (25). Consistent and precise data is an essential measurement for evaluating the incidence, prevalence, and burden of disease at national and international levels (5). Developing an MDS is an important prerequisite for gathering standard, integrated, and uniform data on a disease (26). MDS provides high-quality information for caregivers by creating a national database (20).

Unfortunately, our review has shown that there is no specific registry for regular data collection of KD patients. Therefore, the minimum data set was designed in this study, and as a result, the KD National Registration System of Iran is the first attempt to collect comprehensive data on KD in the world.

Each MDS possesses diverse data elements containing demographic, administrative, and clinical data such as health status, procedures, medications, treatments, and outcomes (20, 27). Based on the results of our study, similar to other disease registration systems in the world, the necessary data elements required for designing a national registry for KD can be classified into administrative and clinical groups.

Some of our data elements, like some demographic and therapeutic procedures data, are common with other registries and guidelines for KD diagnosis, management, and treatment in the world and especially with what had been defined in KD case report forms in the USA, Korea, and Hong Kong (28–30). Apart from the common data elements, some data elements were considered desirable according to the regional information needs of physicians, other caregivers, and researchers. Accordingly, these data elements will be specific to the National Registration Program of KD in Iran, including the history of the post-Kawasaki disease, history of vasculitis and autoimmune disease, history of contact with certain detergents in recent months, history of allergies, and history of vaccination. Also, the minimum data set designed in this investigation is more comprehensive in many areas, including laboratory and paraclinical tests, than the reviewed case report forms and existent guidelines.

The authors' review indicates despite the notable increasing incidence of disease, there is no KD-related registry in the world. Hence, the result of this study is the first effort to design a national KD registry. It is expected that defining precise, integrated, and comprehensive data elements and developing the IR-KDMDS will lead to effective disease management and thus improve the quality of care and, consequently, improve public health.

The momentous strength of this project is the process of determining the MDS. IR-KDMDS is the outcome of the brainstorming, negotiation, and consensus of experts in several fields related to KD. Hence, it has been comprehensively containing all aspects of diagnosis, treatment, and management of KD.

## Conclusion

This article provides the first comprehensive MDS for developing a systematic KD registry in the world. Therefore, it is useful for designing and implementing a nationwide registry with the purpose of gathering all desired data from all patients across the country for determining the burden of disease and making decisions for effective management of the disease.

## Data Availability Statement

The original contributions presented in the study are included in the article/Supplementary Material, further inquiries can be directed to the corresponding author/s.

## Author Contributions

MR designed the project. ZQ, LS, and MN performed the project. ZQ performed the analytic calculations. ZQ and MR wrote the manuscript. All authors contributed to the article and approved the submitted version.

## Conflict of Interest

The authors declare that the research was conducted in the absence of any commercial or financial relationships that could be construed as a potential conflict of interest.

## Publisher's Note

All claims expressed in this article are solely those of the authors and do not necessarily represent those of their affiliated organizations, or those of the publisher, the editors and the reviewers. Any product that may be evaluated in this article, or claim that may be made by its manufacturer, is not guaranteed or endorsed by the publisher.
