# Supplementary material for: Developing a National Minimum Data Set for Kawasaki Disease Registry in Iran
Source: Front Pediatr. 2022 Feb 28;10:834306. doi: 10.3389/fped.2022.834306 (PMC8919186; doi:10.3389/fped.2022.834306)
Supplement: Supplementary file 1 [file Data_Sheet_1.PDF]

The list of final articles which were retrieved in the systematic review.

1- McCrindle BW, Manlhiot C, Newburger JW, Harahsheh AS, Giglia TM, Dallaire F, Friedman K, Low T, Runeckles K, Mathew M, Mackie AS. Medium-term complications associated with coronary artery aneurysms after Kawasaki disease: a study From the International Kawasaki Disease Registry. Journal of the American Heart Association. 2020 Aug 4;9(15):e016440.

2- Elias MD, McCrindle BW, Larios G, Choueiter NF, Dahdah N, Harahsheh AS, Jain S, Manlhiot C, Portman MA, Raghuveer G, Giglia TM. Management of multisystem inflammatory syndrome in children associated with COVID-19: a survey from the International Kawasaki Disease Registry. Cjc Open. 2020 Nov 1;2(6):632-40.

3- Sung RY, Ng YM, Choi KC, Mok GC, Cheng YW, Ho MH, Hong Kong Kawasaki Disease Study Group. Lack of association of cervical lymphadenopathy and coronary artery complications in Kawasaki disease. The Pediatric infectious disease journal. 2006 Jun 1;25(6):521-5.

4- Durongpisitkul K, Sangtawesin C, Khongphatthanayopthin A, Panamonta M, Sopontammarak S, Sittiwangkul R, Pongpanich B. Epidemiologic study of Kawasaki disease and cases resistant to IVIG therapy in Thailand. Asian pacific journal of allergy and immunology. 2006 Mar 1;24(1):27.

5- Uehara R, Belay ED. Epidemiology of kawasaki disease in Asia, Europe, and the United States. Journal of epidemiology. 2012 Mar 5:1201310285-.

6- Coustasse A, Larry J, Lee D. Can Kawasaki disease be managed?. The Permanente Journal. 2012;16(2):70.

7- Wolff AE, Hansen KE, Zakowski L. Acute Kawasaki disease: not just for kids. Journal of general internal medicine. 2007 May;22(5):681-4.

8- Salo E, Griffiths EP, Farstad T, Schiller B, Nakamura Y, Yashiro M, Uehara R, Best BM, Burns JC. Incidence of Kawasaki disease in northern European countries. Pediatrics International. 2012 Dec;54(6):770-2.

9- Park YW, Han JW, Park IS, Kim CH, Cha SH, Ma JS, Lee JS, Kwon TC, Lee SB, Kim CH, Lee HJ. Kawasaki disease in Korea, 2003–2005. The Pediatric infectious disease journal. 2007 Sep 1;26(9):821-3.

10- Loke YH, Berul CI, Harahsheh AS. Multisystem inflammatory syndrome in children: is there a linkage to Kawasaki disease?. Trends in cardiovascular medicine. 2020 Jul 20.

11- Chen JJ, Ma XJ, Liu F, Yan WL, Huang MR, Huang M, Huang GY, Shanghai Kawasaki Disease Research Group. Epidemiologic features of Kawasaki disease in Shanghai from 2008 through 2012. The Pediatric infectious disease journal. 2016 Jan 1;35(1):7-12.

12- Du ZD, Zhao D, Du J, Zhang YL, Lin Y, Liu C, Zhang T, Beijing Kawasaki Research Group. Epidemiologic study on Kawasaki disease in Beijing from 2000 through 2004. The Pediatric infectious disease journal. 2007 May 1;26(5):449-51.

13- Satou GM, Giamelli J, Gewitz MH. Kawasaki disease: diagnosis, management, and long-term implications. Cardiology in Review. 2007 Jul 1;15(4):163-9.

14- Singh S, Vignesh P, Burgner D. The epidemiology of Kawasaki disease: a global update. Archives of disease in childhood. 2015 Nov 1;100(11):1084-8.

15- Bronstein DE, Dille AN, Austin JP, Williams CM, Palinkas LA, Burns JC. Relationship of climate, ethnicity and socioeconomic status to Kawasaki disease in San Diego County, 1994 through 1998. The Pediatric infectious disease journal. 2000 Nov 1;19(11):1087-91.

16- Freeman AF, Shulman ST. Recent developments in Kawasaki disease. Current opinion in infectious diseases. 2001 Jun 1;14(3):357-61.

17- Lin MC, Lai MS, Jan SL, Fu YC. Epidemiologic features of Kawasaki disease in acute stages in Taiwan, 1997–2010: effect of different case definitions in claims data analysis. Journal of the Chinese Medical Association. 2015 Feb 1;78(2):121-6.

18- Witt MT, Minich LL, Bohnsack JF, Young PC. Kawasaki disease: more patients are being diagnosed who do not meet American Heart Association criteria. Pediatrics. 1999 Jul 1;104(1):e10-.

19- Singh S, Jindal AK, Pilania RK. Diagnosis of Kawasaki disease. International journal of rheumatic diseases. 2018 Jan;21(1):36-44.

20- Sosa T, Brower L, Divanovic A. Diagnosis and management of Kawasaki disease. JAMA pediatrics. 2019 Mar 1;173(3):278-9.

21- *McCrindle BW, Rowley AH, Newburger JW, Burns JC, Bolger AF, Gewitz M, Baker AL, Jackson MA, Takahashi M, Shah PB, Kobayashi T. Diagnosis, treatment, and long-term management of Kawasaki disease: a scientific statement for health professionals from the American Heart Association. Circulation. 2017 Apr 25;135(17):e927-99.*

22- Rowley AH. Incomplete (atypical) Kawasaki disease. The Pediatric infectious disease journal. 2002 Jun 1;21(6):563-5.

23- Senzaki H. Long-term outcome of Kawasaki disease. Circulation. 2008 Dec 16;118(25):2763-72.

24- Chaiyarak K, Durongpisitkul K, Atta T, Soongswang J, Laohaprasitiporn D, Nana A. Clinical manifestations of Kawasaki disease: what are the significant parameters?. Asian Pacific journal of allergy and immunology. 2009 Jun 1;27(2-3):131.

25- Gorrab AA, Fournier A, Bouaziz AA, Spigelblatt L, Scuccimarri R, Mrabet A, Dahdah N. Incidence rate and epidemiological and clinical aspects of kawasaki disease in children of maghrebi origin in the Province of Quebec, Canada, compared to the country of origin. Global pediatric health. 2016 Feb 16;3:2333794X16630670.

26- Jakob A, Whelan J, Kordecki M, Berner R, Stiller B, Arnold R, von Kries R, Neumann E, Roubinis N, Robert M, Grohmann J. Kawasaki disease in Germany. The pediatric infectious disease journal. 2016 Feb 1;35(2):129-34.

27- Pinto FF, Laranjo S, Mota Carmo M, Brito MJ, Cruz Ferreira R. Twelve Years of Kawasaki Disease in Portugal. The Pediatric infectious disease journal. 2017 Apr 1;36(4):364-8.

28- Fernandez-Cooke E, Barrios Tascón A, Sánchez-Manubens J, Antón J, Grasa Lozano CD, Aracil Santos J, Villalobos Pinto E, Clemente Garulo D, Mercader Rodríguez B, Bustillo Alonso

M, Nuñez Cuadros E. Epidemiological and clinical features of Kawasaki disease in Spain over 5 years and risk factors for aneurysm development.(2011-2016): KAWA-RACE study group. PLoS One. 2019 May 20;14(5):e0215665.

29- Mastrangelo G, Cimaz R, Calabri GB, Simonini G, Lasagni D, Resti M, Trapani S. Kawasaki disease in infants less than one year of age: an Italian cohort from a single center. BMC pediatrics. 2019 Dec;19(1):1-7.

30- Ramphul K, Mejias SG, Joynauth J. Kawasaki disease among children in the United States. Reumatologia. 2019;57(4):253.

31- Ng YM, Sung RY, So LY, Fong NC, Ho MH, Cheng YW, Lee SH, Mak WC, Wong DM, Yam MC, Kwok KL. Kawasaki disease in Hong Kong, 1994 to 2000.

32- Huang YH, Lin KM, Ho SC, Yan JH, Lo MH, Kuo HC. Increased incidence of Kawasaki disease in Taiwan in recent years: a 15 years nationwide population-based cohort study. Frontiers in pediatrics. 2019 Mar 29;7:121.

33- Nakamura Y, Aso E, Yashiro M, Tsuboi S, Kojo T, Aoyama Y, Kotani K, Uehara R, Yanagawa H. Mortality among Japanese with a history of Kawasaki disease: results at the end of 2009. Journal of epidemiology. 2013 Nov 5;23(6):429-34.

34- Siadati A, Sabouni F. Kawasaki Disease (KD) in Iran: A Report of 85 Cases. Journal of Comprehensive Pediatrics. 2007 Dec 31;1(2):9-12.

35- Makino N, Nakamura Y, Yashiro M, Sano T, Ae R, Kosami K, Kojo T, Aoyama Y, Kotani K, Yanagawa H. Epidemiological observations of Kawasaki disease in Japan, 2013–2014. Pediatrics International. 2018 Jun;60(6):581-7.

36- Sharif M.R., Iranfar M. Surveying The Related Factors In Kawasaki Disease In Children Hospitalized In Shaheed Beheshti Hospital (Kashan) And Tehran Children's Center, 1995-2000. 2002:6 (22): 39-44.

37- SABRI MR, RAHIMI H, MOUSAVI SM, ABEDINI A, ROOMIZADEH P, MAHMOUDI F, HAGHIGHIZADEH E. Evaluation of early-and late-onset cardiovascular disorders and related risk factors in children with Kawasaki disease.

38- Shamsizadeh A, Kajbaf TZ, Razavi M, Cheraghian B. Clinical and epidemiological characteristics of Kawasaki disease. Jundishapur journal of microbiology. 2014 Aug;7(8).

39- Yazdi AK, Akbariasbagh P, Aghighi Y, Raeeskarami SR, Toomaj K, Heidari S, Alamdari S, Sahebi L. Impact of Early Treatment With High-Dose Intravenous Immunoglobulin on Incidence of Kawasaki Disease Complications in Iranian Children. Journal of Family and Reproductive Health. 2021 Nov 28:242-7.

40- Soleimani G, Sadeghi Bojd S, Tajik M, Shafighi Shahri E, Rashidi S. Paraclinical evolutions regarding liver and renal abnormalities of Kawasaki disease in the Southeast of Iran. J Compr Ped. 2014 Feb 27;5(1):15777.

41- Moradinejad MH, Kiani A. Kawasaki disease in 159 Iranian children. *Iranian Journal of Pediatrics . 2007: 17 (3); 241-246.*

42- Rezaee MS, Ghafari J, Zamani H, Fattahi S. Incomplete and atypical presentation of Kawasaki disease: a report of four cases and review of literature. Journal of Mazandaran University of Medical Sciences. 2011 Dec 10;21(85):166-72.

43- H Mahmoudzadeh, AA Nikibakhsh, SS Gheibi, A Aghayar Makoui. A SURVEY ON KAWASAKI DISEASE IN IMAM KHOMEINI HOSPITAL, URMIA. Stud Med Sci. 2008; 19 (3) :236-241.

44- Moghadam AR, Hoseinzadeh M. Study of clinical and laboratory findings in children with Kawasaki disease hospitalized at Ahvaz Abuzar and Golestan hospitals. Scientific Medical Journal (AJUMS). 2011;9(6):543-52.

45- Marrani E, Burns JC, Cimaz R. How should we classify Kawasaki disease?. Frontiers in immunology. 2018 Dec 14;9:2974.

46- Tizard EJ. Complications of Kawasaki disease. Current paediatrics. 2005 Feb 1;15(1):62-8.

47- Gordon JB, Burns JC. Management of sequelae of Kawasaki disease in adults. Global cardiology science & practice. 2017 Oct 31;2017(3).

48- Kim KY, Kim DS. Recent advances in Kawasaki disease. Yonsei medical journal. 2016 Jan 1;57(1):15-21.

49- Newburger JW, Takahashi M, Burns JC. Kawasaki disease. Journal of the American College of Cardiology. 2016 Apr 12;67(14):1738-49.

50- Piram M, Koné-paut I, Djemoui A, Dallochio A, Lechevalier P, Gajdos V, Launay E, Eyssette-Guerreau S, Ballot C, Michelet I, Poignant S. AB0754 Kawanet: The french registry for kawasaki disease (KD). Annals of the Rheumatic Diseases. 2013 Jun 1;71(Suppl 3):681-.

51- Pilania RK, Bhattarai D, Singh S. Controversies in diagnosis and management of Kawasaki disease. World journal of clinical pediatrics. 2018 Feb 8;7(1):27.
